# An In Toto Approach to Radon Dispersion Modelling from a South African Gold Mine Tailings

**DOI:** 10.3390/ijerph19138201

**Published:** 2022-07-05

**Authors:** Frank Komati, Martin Ntwaeaborwa, Rian Strydom

**Affiliations:** 1 Department of Mathematical and Physical Sciences, Central University of Technology, Private Bag X 20539, Bloemfontein 9300, South Africa; 2 Faculty of Science, School of Physics, University of the Witwatersrand, Private Bag 3, Braamfontein 2050, South Africa; martin.ntwaeaborwa@wits.ac.za; 3 Parc Scientific, Cresta 2118, South Africa; parc@axxess.co.za

**Keywords:** radon, tailings dams, dispersion modelling, wake effect, radon transport

## Abstract

The USA Environmental Protection Agency’s (EPA) Industrial Source Complex Short Term 3 (ISCST3) dispersion modelling code was used to evaluate radon transport and the effects of local variations around tailings dam using a Gaussian plume model. The tailings dam was modelled as point, flat ground and top level, total emitting surface area (true geometry) and volume source geometries. The true area geometry was considered as the baseline source geometry. To improve the accuracy of the model predictions as compared to traditional approaches, the true geometry area source term was corrected to account for cracks and fissures on the tailings and the geometry of tailings dam was modelled by considering all emitting surfaces as sources. Compared to the baseline, the model overpredicted the flat ground area source by up to 274% and underpredicted the top-level area source by up to 50%. The volume emission source was overpredicted by up to 300% in 60% of the modelling runs and underpredicted by 55% in 40% of the volume model runs. While the top-level area source term produced lower concentrations at near-field ground-level receptors, accounting for the wakes effect increased the radon concentrations from the top-level area source of the tailings dam by up to 239%. From the modelling results, the highest concentration predicted by the model from the true geometry source was found to be 0.843 Bq m^−3^, which corresponds to the dose of 0.012 mSv/y to the public due to radon from the tailings. This value is less than the 1 mSv/y dose constraint stipulated by the National Nuclear Regulator.

## 1. Introduction

The emission of radon (^222^Rn) from a gold mine tailings dam containing radium (^226^Ra) is a potential source of environmental contamination and therefore a possible health hazard to the nearby population. Radon, an inert gas originating as a product of radioactive decay from radium in the tailings material, diffuses to the surface of the dam and escape to the atmosphere, thereby dispersing into the surroundings. When inhaled in large amounts, radon’s alpha emitting short-lived progeny ^218^Po and ^214^Po may lead to lung cancer [1]. This calls for the assessment of atmospheric radon around such facilities, especially when they are near residential areas or sensitive receptors. Consequently, the estimation of radon concentrations at some distances from a source becomes critical for regulatory health risk assessments.

According to [2], South Africa’s deep underground gold reserves contain low-grade uranium. Given that gold is significantly less abundant in the ore than uranium, with a gold-to-uranium ratio ranging from roughly 1:10 to 1:100 [3], significant amounts of uranium and other undesired radioactive materials have been brought to the surface and disposed of as waste on tailings dams by gold mining operations. The presence and vast distribution of uranium and its decay products from tailings constitute a significant source of environmental radiation [4,5] which has the potential to fractionally elevate public exposure beyond recommended levels. Hence, it is plausible to believe that some South African gold mines have contributed either directly or indirectly to uranium pollution and radioactivity elevation, including radon concentrations in areas near uranium producing mines [6,7].

The atmospheric introduction and dispersion of radon from the tailings dam is primarily influenced by convective diffusion, bulk air movements and transport which in turn, are controlled by combined effects of air turbulence, release concentration and height, wind strength, speed and direction, vertical temperature gradient, atmospheric stability etc. Once emitted, radon will mix with the ambient air, causing it to disperse and its concentration will decrease with distance from the source [8,9].

Air dispersion models are designed and applied to simulate and forecast the fate and transport of atmospheric emissions of pollutants from industrial sources such as tailings dams. Previously, simulations of outdoor atmospheric dispersion of radon were carried out with (i) Gaussian models in radioactive waste disposal facilities [10,11] and uranium tailings [12,13,14]; (ii) Box model in in-situ soil measurements [15]; (iii) Lagrangian model in transport station [16] and (iv) Computational Fluid Dynamics (CFD) model in uranium mine shaft ventilation [17,18]. In South Africa, as part of radon monitoring routine, the environmental radon contribution from gold mine tailings dams has been the basis of various studies and regulatory programmes because of gold mining activities. However, there is relatively limited literature on the use of dispersion models to forecast and quantify radon contributions from tailings dam sites. The few available reports are mostly the unpublished radiological safety assessment technical reports from environmental radioactivity assessment impact studies relating to uranium, gold, and other mining industries [19,20,21], which are in the proprietary rights of regulated mining companies, and basic modelling data from an unpublished radon monitoring thesis [22]. In all of these reports and studies, short term Gaussian models were used to monitor and predict radon concentrations on and off site in the gold mining sector. Notable features from these tailings dam dispersion modelling studies revealed the following:

1. many of these studies assumed uniform pollutant emissions from the dam, in most cases a unit emission rate [16,23], thereby either overestimating or underestimating the source input values. Changes in emissions caused by slope areas, gulleys and cracks within the tailings surface were not considered;
2. all of the area sources were modelled as ground-level flat sources, ignoring the influence of dam height on the near ground receptors. This led to the expectation of a higher estimate of the predicated radon concentration for near ground receptors;
3. wake effects, which have the potential to significantly increase radon levels at near ground receptors, were not considered in all the studies;
4. for 3-dimensional sources, a single volume source is not always suitable when used in ISCST3. In the case of tailings dams, their enormous size and design may require dividing one volume source into multiple volumes, which can have unintended consequences of altering the true size of the emitting source; and
5. from a dispersion modelling viewpoint, tailings dams can be categorised as non-point sources because of their large geometrical structures. However, using non-point sources such as area and volume sources to model dispersed pollutant from large structures such as tailings dams and big buildings have been marred by poor source characterisation due to inadequately defined physical features and highly unreliable emission rates [24]. Therefore, it becomes challenging to model and properly quantify complex geometries of regulated radon sources such as tailings dams near or at ground level.

It is apparent from the studies that there is lack of proper quantification of the source term model input values used within the air dispersion model when simulating radon from tailings dams given that the accuracy of the estimates depends on the reliability of the source-term input data. It is therefore critical to identify and quantify the radon source term as accurately as possible.

In this study, we used the steady-state Gaussian plume Industrial Source Complex Short Term 3 (ISCST3) commercial atmospheric dispersion software package by BREEZE AERMOD GIS Pro (Version 4.0., Trinity Consultants Inc., Dallas, TX, USA, 2002) and local weather data to simulate and calculate radon concentrations from the tailings dam and specified receptor points. The ISCST3, an EPA model designed to reinforce regulatory modelling programs [25], is a steady-state Gaussian plume dispersion model specifically developed for simulation of pollutants due to different sources related to industrial complexes. The ISCST3 has been widely applied in air-quality studies in the past (for example, Refs. [26,27,28,29,30]) and has a good track record of monitoring compliance with air quality standards. The ISCST3 can accommodate the following: point, line, area, and volume sources; dry deposition and downwind depletion of the pollutant, different types of pollutants; terrain adjustments; building downwash; wake effects; source apportionment analyses and plume rise as a function of downwind distance [31]. It can also account for non-reactive pollutants, particulate matter as well as first order radioactive decays [32]. The advantages of ISCST3 over other Gaussian models (AERMOD, ADMS, etc.) are its relative simplicity of operation and its vigorous, robust, and reproducible predictions [33,34]. The greatest advantage for this study is the fact that ISC uses meteorological data from local weather stations whereas AERMOD uses data from mesoscale meteorological models. Local weather data would produce more accurate results on the “micro” scale of this project. In addition, the choice of ISCST3 for this study over the recent EPA approved AERMOD was influenced by the fact that ISCST3 was found to perform better than AERMOD under stable winter conditions [35] and flat simple terrains [9] as has been the case in this study.

To accurately estimate radon source term emissions from the tailings dams, considerations were taken into account for changes in radon emissions from the tailings surface, the geometry of the selected tailings dam and the effects of recirculation behind the trailing edge of the tailings dam (wake effect). This study is part of a broader investigation to validate atmospheric radon dispersion modelling and that study will be referred to often in this document. Therefore, as part of the validation process, we deemed it necessary to appropriately compare the modelled and measured concentrations. The validation study necessitated identification of a tailings dam isolated from other tailings dams or radon sources. For this purpose, an isolated tailings dam in the Free State goldfields was chosen for this study.

## 2. Materials and Methods

### 2.1. Study Area

**Figure 1:**
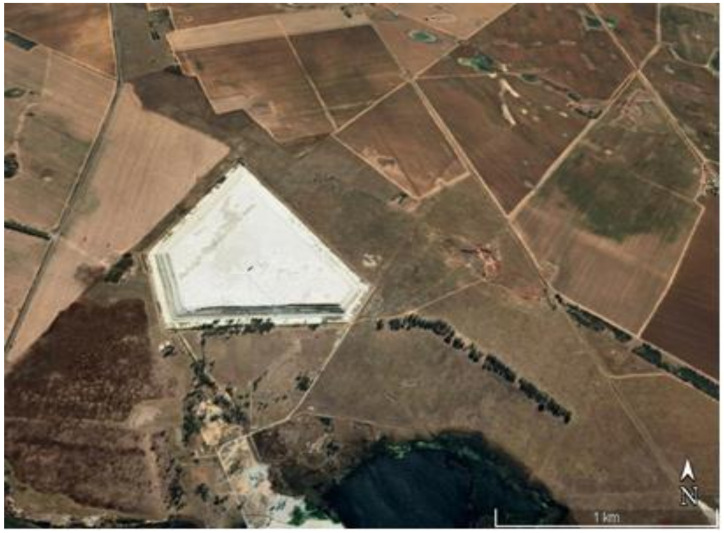
Aerial view of tailings dam (Google Earth^®^).

An isolated and representative tailings dam (Figure 1) located in Lejweleputswa District Municipality, Free State Province, (South Africa) between Odendaalsrus (−27°51′59.99′′ S, 26°40′59.99′′ E) and Allanridge (27°45′15.52″ S, 26°38′37.75″ E) towns was selected for the study. This dry, dormant tailings dam contains gold mine tailings from an old, closed mine shaft located in the southern part of the dam. The tailings dam is bordered by a township to the north, the R30 main road and a vegetation farm to the west, a small pond, and a hostel with occupancy of about two hundred people to the southeast of the tailings. The nearest tailings dam is located approximately 5 km northwest of the tailings dam. This tailings dam is still “active” with wet sludge from the adjacent operational gold mine. Other tailings dams are located at 8.6 km and 16 km, respectively, on the south and south-east side of the tailings. There are no other tailings dams or other man-made sources in the north, north-east and east of the dam.

**Figure 2:**
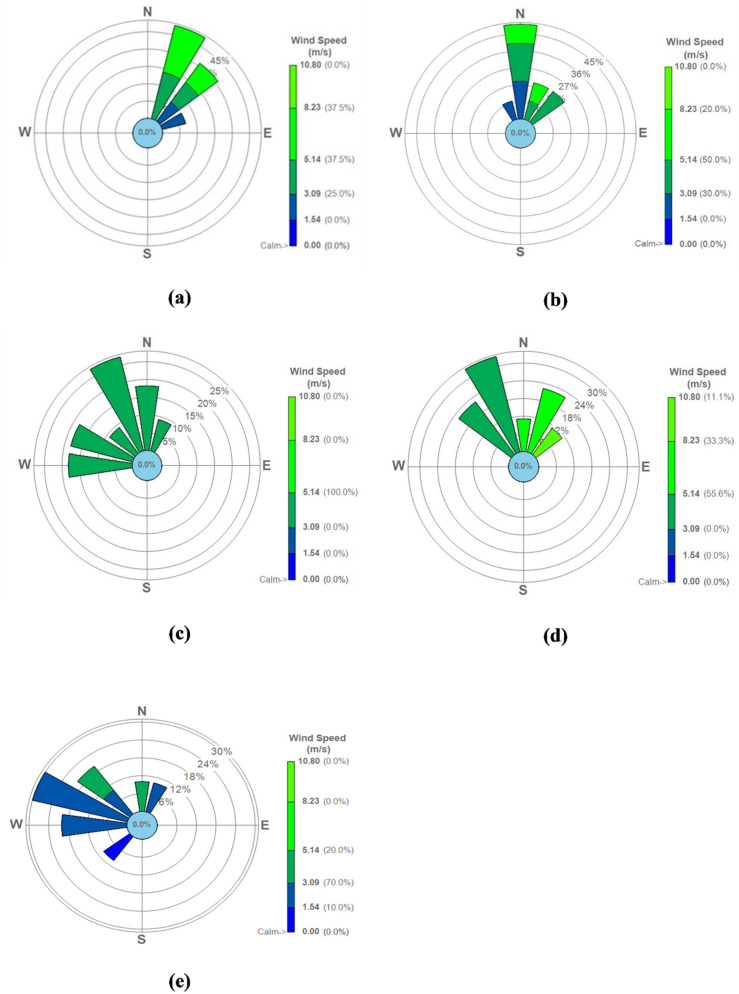
Wind roses for the period 19–27 August 2017: (**a**) day 1(19 August 2017); (**b**) day 2 (20 August 2017); (**c**) day 3 (21 August 2021); (**d**) day 4 (26 August 2021) and (**e**) day 5 (27 August 2021).

To accurately simulate radon dispersion from the tailings dam requires local meteorological data from the study location and period. As a result, the South African Weather Service (SAWS) provided 5-min weather data for the Welkom—Odendaalsrus region from 19 August to 27 August 2017, as the closest station with sufficient and reliable data. The station is located about 20 km in direction south-east from the site. Due to the flat topography of the study area the data is expected to be representative of the site. The meteorological wind roses are depicted in Figure 2 and cover the whole study time domain. During the winter months of June to August, the area was characterised by a dominant average wind from the north, north-east, and north-west. The most common directions were north-east for day 1 (100%) and day 4 (100%), north for day 2 (50%), and north-west for day 3 (100%) and day 5 (90%). Wind speeds for the area varied between 1.5 m/s (day 5) and 10.80 m/s (day 4) while calm (<1.5 m/s) and turbulent (>10.8 m/s) periods occurred for 10% and 11.1% of the time, respectively. High wind speeds were generally related with north-easterly winds, whereas low wind speeds were mostly associated with north-westerly winds. During the data collection period, average daily minimum and maximum temperatures ranged from 9 °C to 20 °C. During this period, there was no rain.

### 2.2. ISCST3 Dispersion Model

Radon gas from radium containing tailings dam was modelled using ISCST3 dispersion model to compute ground-level concentration from point, area, and volume sources. The wind speed was adjusted using a stability-dependent power law and the vertical and lateral dispersion were estimated using the Pasquill–Gifford curves [25]. The dispersion concentration was calculated from the radon flux released from contaminated emission area, or the sum of several areas.

The Plume Rise Model Enhancements (PRIME) algorithm was used to model wake effects. It was developed and incorporated into the ISCST3 to account for two key characteristics: increased plume dispersion coefficients due to wake turbulence, and reduced plume rise due to descending streamlines and increased entrainment in the wake of a structure. Its basis is the ability to model the downwind cavity (near wake) and far wake areas on a three-dimensional scale. The wake effect algorithm is only applicable to point sources; volume or area sources are not incorporated. In addition, the algorithms that model the building wakes require extra input data generated by running the EPA Building Profile Input Program (BPIP) for all of the point sources. The BPIP is used to establish the probability of a point source to be subjected to wake effects due to the surrounding structures [9].

#### ISCST3 Model Input Parameters

Meteorological conditions, topography, receptor coordinates across the modelling area, and source/emission parameters are the primary ISCST3 input requirements for regulatory air quality analysis by the USEPA. In this study, the model was run in concentration mode for all of the sources using the USEPA regulatory default options, 1-h and total period average concentration option, non-regulatory rural dispersion coefficients and the receptor height of 1.5 m. Flat terrain parameters were selected based on the characteristics of the immediate vicinity of the selected tailings dam. The model output predictions were due to specified source emissions from the tailings dam only and did not include background concentrations.

Source/emission input parameters describing the atmospheric release of radon varied according to the type of source. The main inputs from the source/emission options applied in this study were: number of sources (multiple sources from all sides of the tailings); output contribution (combined pollutant concentrations from all sides); source type (point (PRIME), area and volume); height of the tailings dam (30 m); height of the sources (varied from 0 at ground flat surface to 30 m at the top of the dam); area shape (polygon); and output (^222^Radon concentrations in µ Bq m^−3^, which was converted to Bq m^−3^). A steady-state area-weighted emission rate of 0.102 Bq m^−2^ s^−1^ that was experimentally determined by [36] was used for all of the area sources. This value was corrected for discrepancies in the side view area measurements of the tailings dam from the modelling software and Google Earth^®^ (see Section 2.3.1). For the point and volume source emission rates, the area emission source term was converted to assigned units of mass/time (for example, g/s) in accordance with EPA guidance [25].

The ISCST3 was programmed to accept hourly averaged meteorological data to simulate plume transport, rise, diffusion and dispersion. Five-minute weather data from 19–27 August 2017, for the Welkom—Odendaalsrus region obtained from the South African Weather Service (SAWS) with anemometer height of 10 m was manipulated and averaged to hourly data suitable to be used in ISCST3 model. To create the ISCST3 ready files, meteorological data files were processed and formatted as FORTRAN executable ASCII files. Five input meteorological ASCII files for each day of measurement were prepared. A sample file for day 1 (19 August 2017) is shown in Table 1.

From Table 1, the station ID number and the year (0364300; 2017) are clearly designated as the first entries of the ASCII file and subsequent records are identically formatted to represent hourly data for sampling and measurement durations. The prescribed fields in each of these records are year, month, day, hour of day, wind direction (degrees), wind speed (meters per second), temperature (degrees Kelvin), atmospheric stability A (1), B (2), C (3), D (4), E (5), F and G merged (6)) and rural mixing height (meters). The vertical mixing heights were calculated from the Pasquil stability and wind speed values proposed by [37]. Conservative mixing heights corresponding to stability classes of 4, 3, 2 and 1 were used.

**Figure 3:**
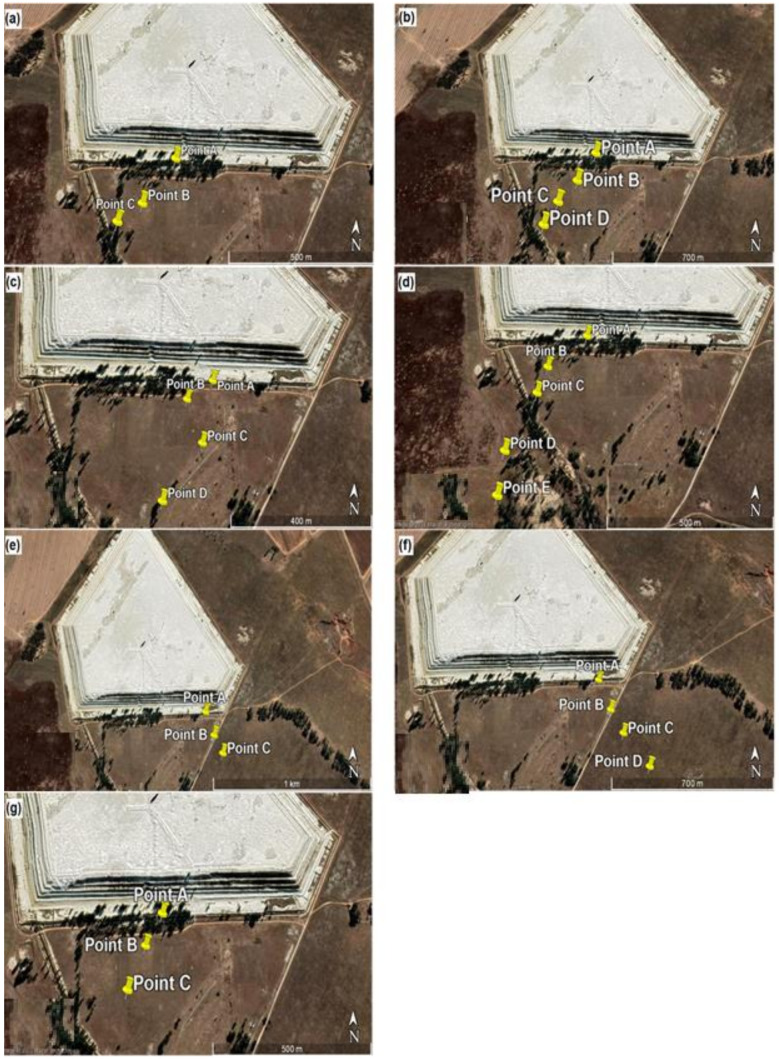
Receptor points for downwind measurements and their positions and distances from the tailings for (**a**) day 1 morning, (**b**) day 1 afternoon, (**c**) day 2 afternoon, (**d**) day 3 morning, (**e**) day 3 afternoon, (**f**) day 4 afternoon and (**g**) day 5 morning.

Discrete Cartesian Coordinates for 26 receptors at the height of 1.5 m above ground were used in the modelling. These 26 receptor points were chosen based on the hourly radon measurements obtained as part the broader study on Validation of radon dispersion modelling from tailings dams. In the approach of following the wind direction, radon was measured downwind in the direction of the wind, starting from a point that was closest to the tailings (source) at time intervals of about 1 h between successive measurements from the base of the tailing. These measurements, taken in the mornings and afternoons over a period of five days consecutively (Figure 3), were carried out with Alpha GUARD PQ2000 PRO pulse ionisation chamber (Saphymo GmbH, Frankfort, Germany) which was operated in flow mode with 10-min cycles.

The closest receptor point to the tailings was chosen as the first receptor point (point A). The second point B was located after about an hour or so by following the direction of the wind some distance from point A. A similar follow the wind approach was applied at points C, D and E located some distances from points B, C and D, respectively. The distances between measurements ranged between 100 m and 200 m, depending on accessibility and suitability. Considering that the multiple aerial and volume source elements making up the dam are not located at the same point, a Cartesian coordinate system in the ISCST3 model was chosen as the most practical. The X and Y coordinates of the receptor were specified from the map view in the downwind direction.

Noting that the ISCST3 modelling solely accounts for radon concentrations in the air that are due to emissions from the tailings dam, modelled radon concentrations were added to the background radon to obtain total radon concentration at each receptor site. It is only then that the predicted and observed concentrations can be appropriately compared. The background concentrations, which account for contributions from natural sources, nearby sources and other unidentified sources excluded in the modelled inventory, were taken as the radon concentrations measured “upwind” to the tailings dam along the same line and wind direction as the “downward” modelling direction. A ± 10^0^ wind variation was allowed since there is no substantial concentration change within this degree range [38]. These background concentrations were added to the modelled concentrations at each receptor location [39].

### 2.3. Modelling Protocol

**Figure 4:**
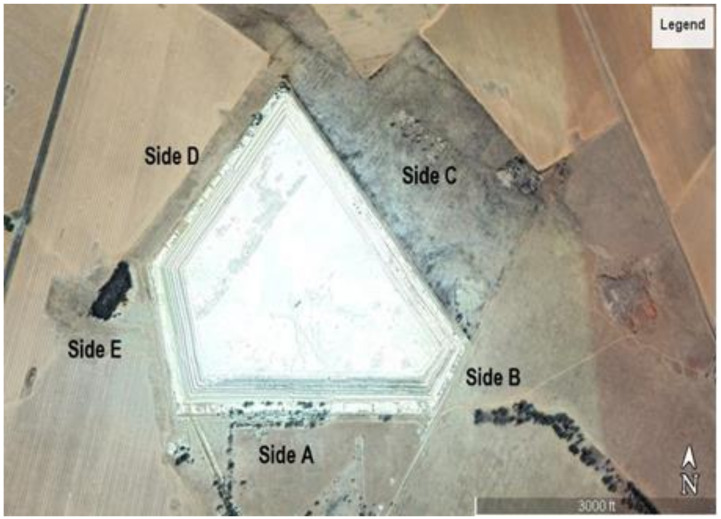
Five (5) sides of the tailings dam.

All of the physical aspects of the source of emission, dimensions, side areas, volume as well as geographical location were used as input data to the model. For the sake of accuracy, clarity and modelling simplicity, the tailings dam was divided into five (5) sides and a top surface. These sides, which will be referred to as sides A–E in various modelling scenarios, are shown in Figure 4.

#### 2.3.1. Measurements of Area and Volume Sources

The map view option of the ISCST3 source input was used to estimate the dimensions of the tailings (and thus the areas and volumes). The tailings dam image was downloaded from Google Earth^®^ and saved as a geo-referenced (JPG) image. The stored image was digitised, imported into ISCST3, and used as a base map in the Map view (GIS) of the ISCST3. The image coordinates were specified by setting both the X and Y coordinates at the south-west corner of the image and the associated Easting X and Northing Y coordinates at (0:0). To ensure that the image size corresponded to the scale of the modelling domain within the BREEZE ISC, the distance scale was set by selecting any two points on the map and specifying the distance between those two points.

**Figure 5:**
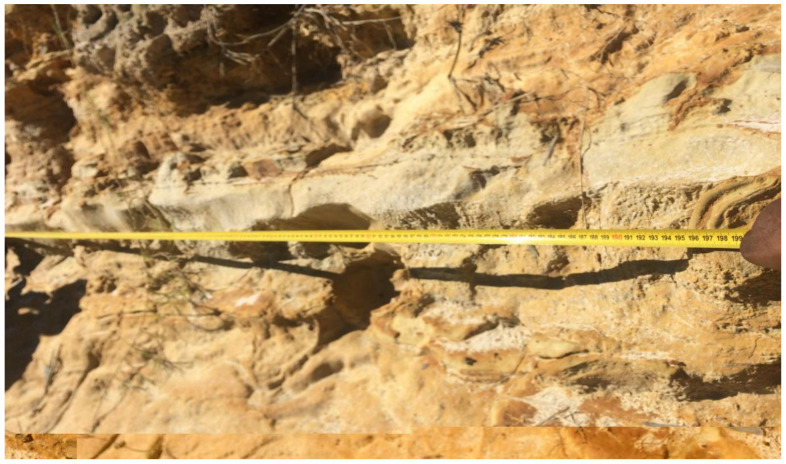
The side view surface of the dam showing cracks and fissures.

All of the area and volume measurements used in the modelling were taken from Google Earth^®^ and BREEZE software (Trinity Consultants. Inc., Dallas, TX, USA). These measurements assumed that the sides of the dam were smooth surfaces. However, the lateral surfaces of the dam have developed fractures and fissures due to aging and years of erosion, resulting in rough structures with grooves which represent a larger surface area than the projected one as illustrated in Figure 5. While the emission rates per unit surface area are likely to be the same, the larger surface suggests a higher overall emission from the side areas than the flat top surface [29].

To obtain more accurate measurements of the side view surface areas, and to consider the effects of the cracks and grooves on the emission rates, two representative measurements of length were taken on a two-meter strip at each of the five sides of the dam. The first measurement was made across a two-meter distance with a measuring tape. A string was used to take the second measurement, which covered the entire length of the surface, including the insides of the cracks, up to the two-meter mark measured with the tape. All Google Earth^®^ measured side view area values and thus the emission rates were corrected using the average of the five string measurements from each of the dam’s five sides. The measurements and the correction factors are given in Table 2.

The emission rates for the side-view areas were corrected for the differences between the measured area (BREEZE) and the true areas (corrected terrain areas using Google Earth^®^) using the relation:

(1) $$E\;\left(\mathrm{corrected}\right)=\;E\;\left(\mathrm{measured}\right)\times \frac{\mathrm{True}\;\mathrm{area}\;\left(\mathrm{terrain}\right)}{\mathrm{Measured}\;\mathrm{area}\;\left(\mathrm{BREEZE}\right)}\;$$

### 2.4. Modelling Scenarios and Analysis

In this study, we postulated that the emissions from the true total surface area of the dam, including all of the source area elements in their true positions, should be the most realistic and accurate modelling option. However, several other geometries have been used in literature with different results. These different geometry scenarios were studied and applied in a series of modelling scenarios using the ISCST3 for non-point sources (area and volume sources) and ISCST3-PRIME for virtual point sources (to account for wake effect) and the results from different scenarios were compared. Modelled concentrations were calculated for 1 h averaging periods. The basic source term settings and modelling approaches that were applied in this study are described below.

#### 2.4.1. Scenario 1: Total Radon Emitting Surface Area (True Geometry)

**Figure 6:**
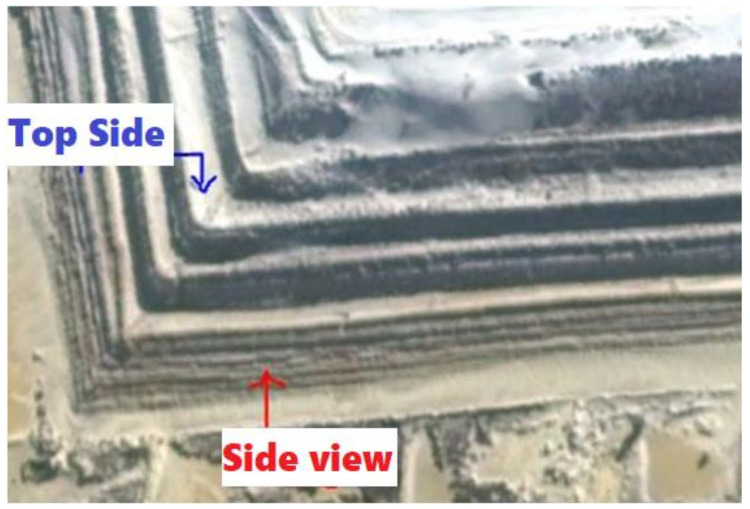
A close-up view of the segments of the side of the dam.

In this emission scenario, the tailings dam was modelled as an area source by considering the total true emitting surface area of the dam. The dam’s whole geometry and dimensions were accounted for, including the slopes on the sides (35°), the height (30 m), the top surface layer, cracks, gulleys and fissures on the side walls of the dam. To model the dam as accurately as possible, the dam was further sub-divided into multiple areas to account for different dimensional variations at each side area. Each of the five sides was divided into ten area segments in accordance with the dam’s design (steps or benches); that is, five top views and five side views. This is illustrated in Figure 6.

**Figure 7:**
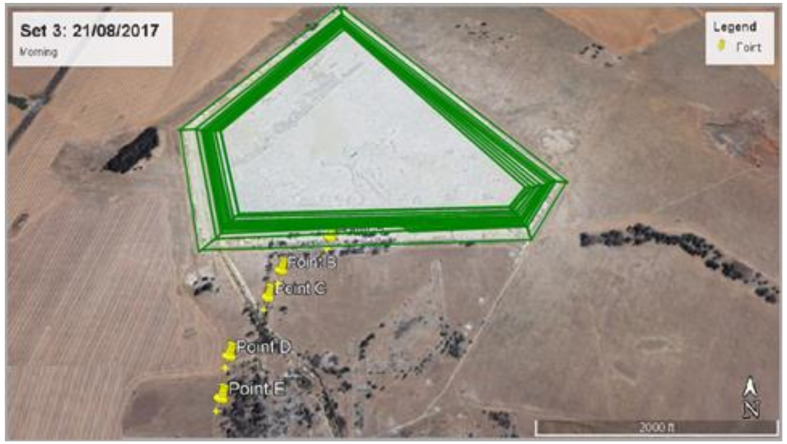
A map view of the total area source from ISCST3.

The areas of each segment shown in Figure 6 were measured as polygon areas in the model objects option of the ISCST3. The emission heights ranged from 0 m at ground level to 30 m at the top of the dam, depending on the area segment. A total of 51 area source elements, comprising the sum of all the areas of each segment, including the top side of the dam, were used as source inputs in the model. A map view of the total area source, including all sides from the ISCST3 is shown in Figure 7.

**Figure 8:**
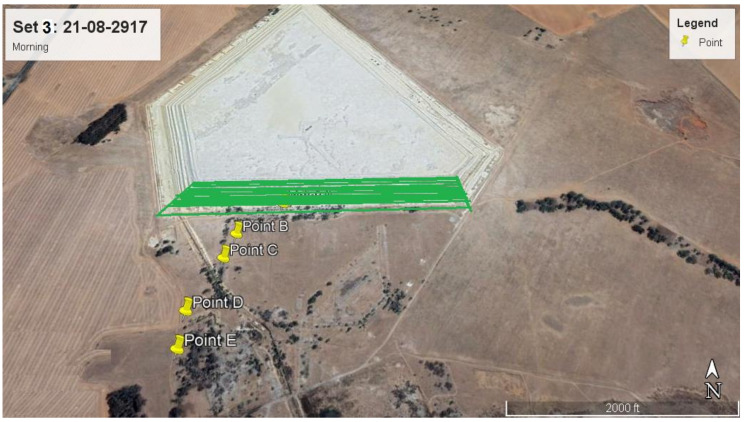
Modelling protocol for day 3 (side A).

A sensitivity analysis was carried out by separately modelling radon contribution from each of the five sides of the tailings dam to establish the impact of individual sides of the dam towards incremental radon concentration at different discrete receptor points downwind. Moreover, 2 representative days (day 3 morning and day 4 afternoon) were chosen for this purpose. Each side was modelled as multiple area source elements as described in scenario 1 and their individual contributions were compared. An example of the modelling protocol for day 3 (side A) is illustrated in Figure 8.

#### 2.4.2. Scenario 2: Ground Level Flat Area Source (Flat Terrain)

**Figure 9:**
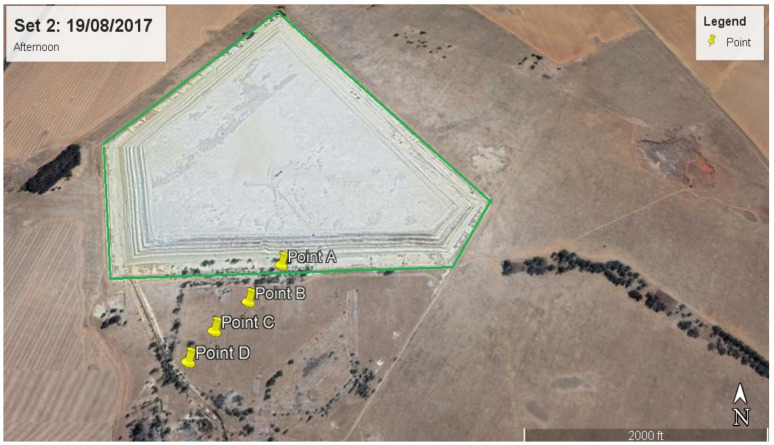
Outline of the ground level surface.

This radon modelling scenario has been applied in numerous studies from tailings dams and is reported extensively [13,19,20,21]. In our case, the tailings dam was modelled as a ground level flat polygon area source by tracing only the outline of the ground level surface from the map view of the ISCST3 as shown in Figure 9. All of the other model input parameters were the same as those mentioned in scenario 1.

#### 2.4.3. Scenario 3: Area Source at the Top of the Dam

**Figure 10:**
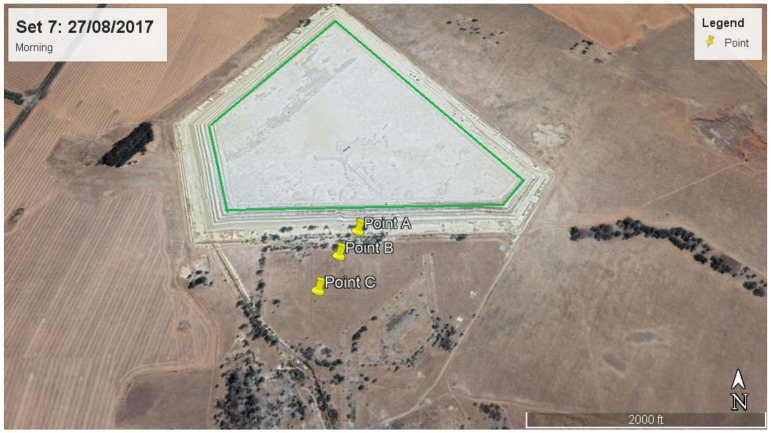
Area source at the top of the dam.

A similar approach to that outlined in scenario 2 was used for the area source at the top of the dam. But in this case, the area source was the dam’s top region, which is 30 m above ground level, as illustrated in Figure 10. The area was calculated and modelled as a polygon area in the ISC source/emission parameters input mode.

#### 2.4.4. Scenario 4: Volume Source

In practice, the volume source modelling approach is commonly applied in modelling emissions escaping from buildings and their dimensions are assigned based on the building size according to the EPA guidelines [9]. Following this practice and given the enormous size and dimensions of the tailings dam, we deemed it necessary to investigate the possibility of modelling the dam as a volume source. To comply with the ISCST3 modelling inputs stipulations [25,32], the ground-level flat area of the dam measured from Google Earth^®^ was considered as the base area of the dam and was found to be 1077,963.06 m^2^. Given the large size of the dam, and for ease of calculations, this ground-level base area was assumed to be a square, which was further subdivided into four (4) equal and smaller cubic volume source elements. Each cube had a base area of 269,490.77 m^2^. As per ISCST3 model source input specifications, the emission rate for any volume source is given in grams per second (g/s). For each of the four volume cubic source elements, the emission rate was calculated from the exhalation flux and the base area relation:

(2) $$\mathrm{Emission}\;\mathrm{rate}\;\left(\frac{g}{s}\right)=\mathrm{flux}\left(\frac{g}{m^{2}s}\right)\times \;\mathrm{area}\;\mathrm{of}\;\mathrm{volume}\;\mathrm{one}\;\mathrm{cube}\;\left(m^{2}\right)\;\;=0.102\;\left(\frac{g}{m^{2}s}\right)\times \;269,490.77{\;m}^{2}\;=2.86\times 10^{4}\;g/s$$

The width of the tailings was taken as the minimum dimension, as such, the model creates a virtual source such that the emissions are released at half the height of the tailings [40]. Given that the height of the dam is 30 m, the initial release height was taken as the centre of the volume at 15 m.

#### 2.4.5. Scenario 5: Modelling for Wake Effects

The wakes effect was modelled using ISCST3 (PRIME) by applying the virtual point source approach proposed by [41]. This method simulates the pollutant plume downwind from an area source as if it came from a point source by replacing the dam’s area source with a virtual point having the same overall strength as the area source. To account for the initial dimensions of the source, the virtual point source was located at some calculated distance upwind (called virtual distance) of the actual area source position. The lateral spread of the plume emanating from the virtual point is then calculated to be comparable to the area source’s width. Virtual point sources were adjusted accordingly for each side of the area source as in scenario 4 by applying Equation (2). The recalculated point source emission rates for the five sides are given in Table 3.

In addition to the emission rates in Table 3, the following input parameters were incorporated for virtual point modelling: temperature (0 K), exit velocity (varied) (m s^−1^), stack height (1 m) and diameter (1 m). The 0 K temperature is the default ISCST3 input emission temperature in the case where the emission temperature is same as the ambient temperature in the meteorological data file, similarly to when a stack is discharging air at the same temperature as the atmosphere. As was the case in this study, the emission temperature from the tailings dam was same as the ambient temperature in the meteorological data file. This required that the emission temperature be set to 0 K in the ISCST3, as this defaults the model to use the ambient temperature, hence the incorporated temperature input parameter of 0 K.The building wake effects can enhance the initial growth of stack plumes. In such instances, lateral (*x_y_*) virtual distance is added by the ISCST3 model to the actual downwind distance x. The lateral virtual distance in kilometres for the rural mode were calculated using the following equation [32]:

(3) $$x_{y}={\left(\frac{\sigma_{y0}}{p}\right)}^{\frac{1}{q}}$$

where *p* and *q*, the wind profile exponents, given in Table 4 are the dimensionless Pasquil stability dependent coefficients obtained from the horizontal plume dispersion coefficients graphs based on Pasquill-Gifford stability classes and *σ_y_*_0_ (m) is the standard deviation of the horizontal wind speed at the source. The values in Table 4 are the ISCSCT3 incorporated default coefficients for modelling in rural mode are functions of stability category and wind speed class. They are used to obtain values for the lateral virtual distance which are used as input to the ISCST3 Gaussian distribution equation to calculate the horizontal dispersion parameters.

The lengths of each side (S) were obtained by computing the square root of each side area in Table 3. The standard deviation of the lateral concentration distribution (*σ_y_*_0_) was calculated by dividing the length of the side of the source S (m) by 4.3 as per [42] recommendation. The results are given in Table 5 below for each side of the tailings dam.

The emission heights were calculated based on the height and the perimeter of the dam as proposed by [32]. For ground based virtual point sources, the emission height was calculated by multiplying the value of the height of the tailings dam (30 m) by 2.5. For virtual point sources that are within the perimeter of the dam, the emission height was calculated by dividing the height of the dam (30 m) by 2.15 [32].

The lateral virtual distances and corresponding emission heights calculated for each side and hour are given in Table 5 for day 2 afternoon downwind run.

## 3. Results and Discussions

ISCST3 dispersion modelling was used to simulate hourly average radon ground level concentrations from the tailings dam at different receptor locations. The results show incremental variations in radon contributions that is attributable to different source geometries.

### 3.1. Modelling Scenarios—No Wake Effect

Table 6 compares the 1-h average radon concentrations predicted by the ISCST3 model from the tailings dam for three area source geometries and a volume source geometry. Other types of sources, such as the wake effect and background concentration, are not included in the modelled results.

Table 6 reveals the model’s sensitivity to site-specific information, particularly source shape and wind direction. Furthermore, the predicted concentrations near the tailings dam (between 0 m and 150 m from the dam) shows high responsiveness to the source height and definition (area or volume). As expected, flat ground-level area source concentrations were the highest of the three area sources and the volume source, whereas top-level area concentrations were the lowest. Since the ISCST3 models area sources as passive emissions, ground-level concentrations are not affected by plume rise and exit velocity, and the plume encounters the ground immediately after release. Given that the receptor heights were chosen at 1.5 m near ground level, generally elevated radon concentrations were observed for flat ground level sources relative to the other modelling source geometries.

Low concentrations from the elevated top level area source 30 m above the ground can be attributed to the certainty that the initial area source emissions at full height will be dispersed above the release height. In this instance, the impact of the plume is not realised on the ground near the tailings, but some distance downwind. At that point, the concentrations are significantly reduced relative to the exit concentrations because of meteorological influences and dilution factors as the plume moves further away from the tailings dam. As shown in Table 6, the emission rates from the elevated top-level source may be similar to those from the flat ground level source, but their impact on the ground level receptor may be significantly lower [43]. The concentration at or around the ground level drops as the source height increases [24].

The volume source utilises source dimensions to determine an initial lateral dimension of a virtual-point source plume at the emission point. This value (width of the source divided by 4.3) is a fraction of the actual measurements of the source. On the other hand, the ISCST3 integrates across the whole extent of the source to treat area sources, thereby giving the area source a much broader plume at the beginning of dispersion and transport.

**Figure 11:**
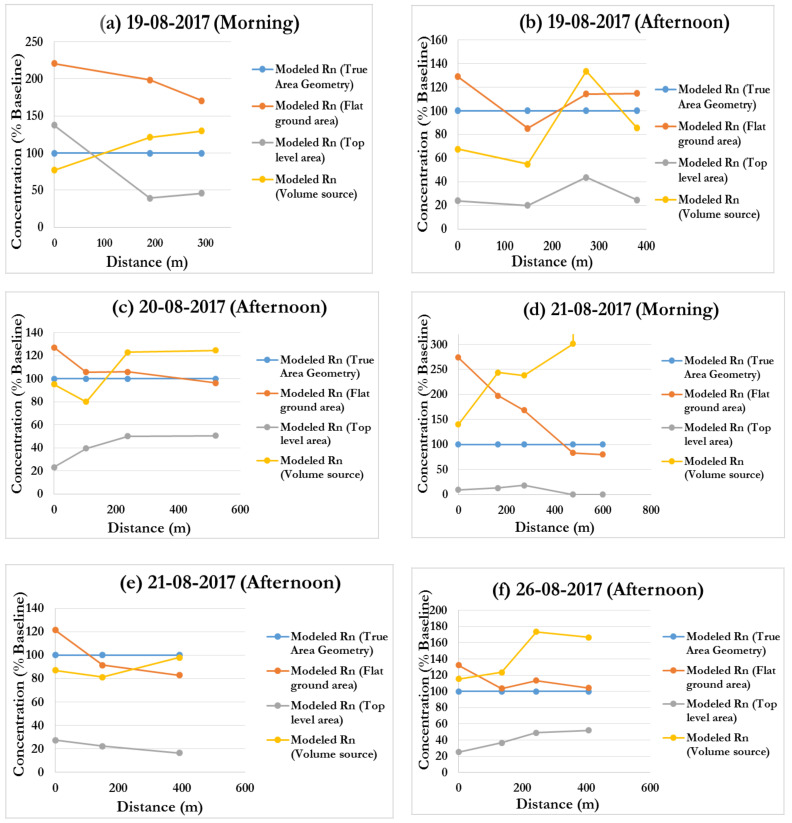

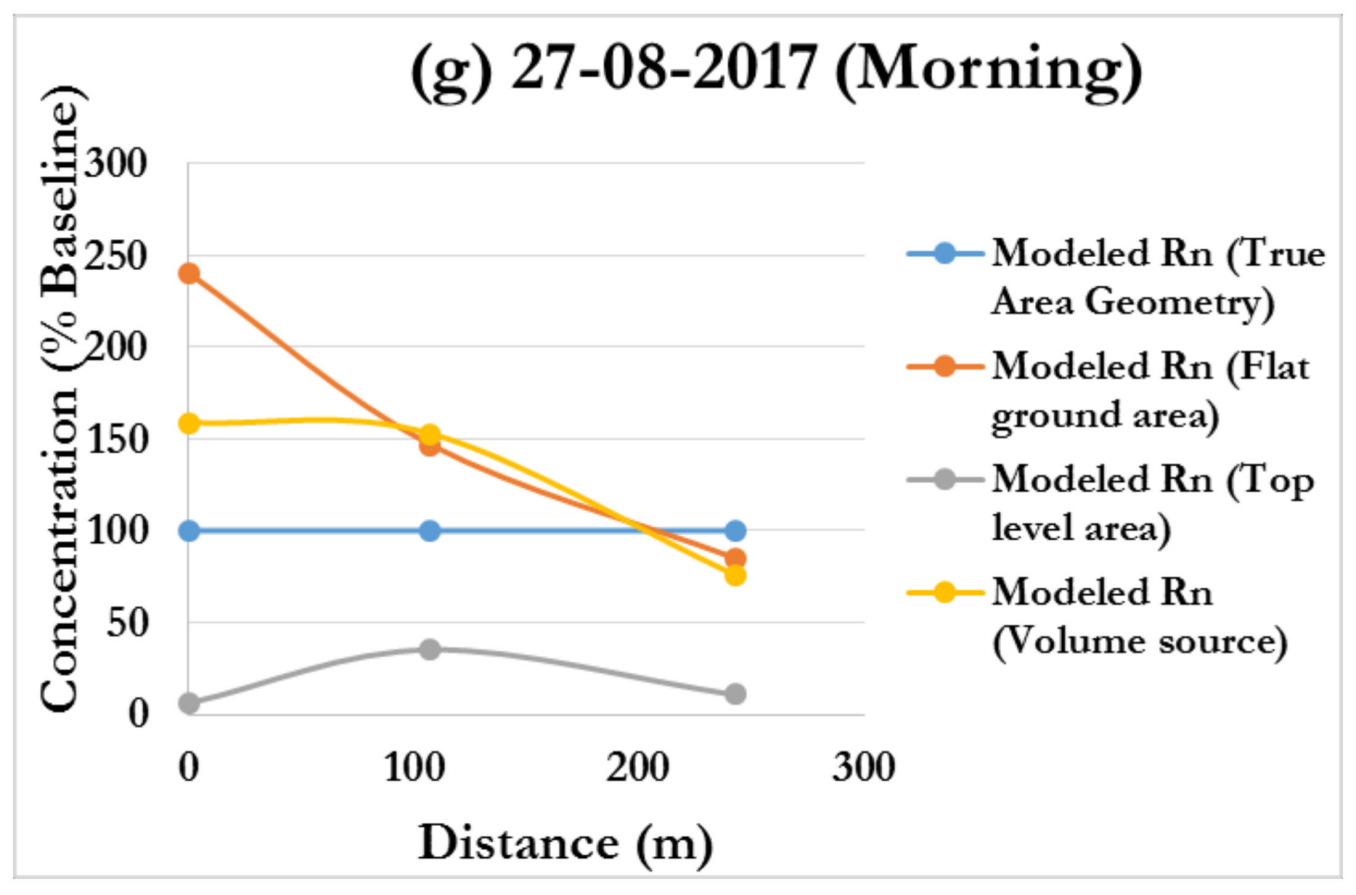
(**a**–**g**). Comparisons of ISCST3 between baseline, flat ground area, top level area and volume sources.

Figure 11a–g present graphical comparisons of the ISCST3 data, with a focus on the influence of different source geometries on receptors. Modelled data were normalized and expressed as a percentage of the baseline (modelling scenario 1, true geometry) which we postulated to be the most accurate representation of the source term.

As expected, the flat ground-level area source concentrations were higher than other source terms. In comparison to the baseline, 74% of the findings for the flat ground source term from a total of 25 simulations yielded overestimated radon concentrations that were 1.04–2.74 times higher than the true geometric area source. When compared to the true geometry area source, the remaining 24% of the flat ground area source underestimated radon concentrations by factors of 0.83–0.96. The highest flat ground-level area values were observed in the mornings along the south-west wind direction, producing high estimations at distances near to the tailings. The biggest overestimation of 2.73 higher than baseline was obtained on day 3 (21 August 2017) morning at nearfield distance very close to the tailings as depicted in Figure 11d.

These phenomena, associated with morning data, can primarily be attributed to radon gas stagnation and recirculation around the areas close to the tailings source which either reduce the air flow velocity or stops the air movement altogether, thereby increasing ground concentration levels in the vicinity of the tailings dam [44] during the calm morning conditions, when the wind speeds and temperatures are low. This explains the observed highest overestimation in day 3 given that predictions were made at the earliest time of the morning (7:39 a.m.) compared to other times, when the conditions for stagnation to occur were highly favourable. On the other hand, large recirculation values are observed during periods of strong turbulent mixing, higher wind speeds and increased temperatures when airborne radon is initially carried away from the tailings source but return later to produce elevated radon concentrations near the source [44]. Hence, high flat ground overestimation factors were observed during the mid-morning and afternoon at receptors close to the tailings. Coincidentally, the highest underestimated concentration of 0.83 times less than the baseline concentration was on the same day afternoon along the south-east wind direction at around 400 m from the tailings (Figure 11e). This point is beyond the recirculation zone and the afternoon conditions favoured strong mixing and vertical inversions. Hence, the presence of low near ground concentrations around that area. Generally, elevated area sources produce lower concentrations at near-field ground level receptors [41,45] as corroborated by results from Figure 11a–g. The model results underestimated the baseline (true geometry area) concentration by between 0.05 and 50.61%.

Volume sources do not model plume rise, as a result, the predicted ground-level concentrations may be overestimated and higher compared to area or point sources at distances less than 0.5 km from the source [24]. From Figure 11, the model over estimated volume concentrations for 60% of the total runs with the biggest overestimated concentration difference of 3 times higher than the baseline concentration at 475.1 m from the tailings on day 3 morning. Conversely, the volume source results underestimated receptor concentrations for 40% of the total runs, with the highest underestimated concentration of 0.55 times less than the baseline concentration on day 1 afternoon at 148.3 m from the tailings dam.

### 3.2. Sensitivity Analysis: Individual Side Modelling

The area emission rate and the downstream radon migration from an area source element on the side of the dam to the receptor determine how much each side of the dam contributes to the overall radon concentration at the receptor point downwind. Table 7 and Table 8 present the modelled radon concentrations and percentage contributions from each of the 5 sides for day 3. Similarly, Table 9 and Table 10 presents concentrations and percentage contributions from each of the 5 sides at each receptor for day 4.

#### 3.2.1. Day 3 Morning

Wind direction on day 3 morning varied with time between NNE (13.67°) and NNW (332.5°) from receptor A to receptor E with the deviation angle between NNE (13.67°) and NNW (332.5°) of 41.17° during sampling periods. Under these conditions, side A was the predominant radon contributor at all of the receptor points, with radon levels dropping as the distance from receptor A to receptor E increased.

From Table 7 and Table 8, it can be shown only three sides contributed to the radon concentrations in receptors A, B, and C. At these three receptor locations, neither side B nor side E contributed anything. Side A (81%) was the most important contributor at receptor A, followed by side C (18.97%) and lastly side D (0.13%). At receptor B, side A’s contribution was reduced from 81% in receptor A to 64.7%, while side D was the second largest contributor at 35.27%, and side C was the least contributor with 0.03%. For point C, the biggest contributor was side A (60.46%), followed by side D (39.4%), and finally side C (0.14%).

At receptors D and E, a distinct pattern was observed. The concentrations at these receptors were extremely low, with side E being the predominant contributor (92.14% and 95.97% for receptor D and E, respectively). With 5.24 percent, side A was the second largest contributor for point D, while side D was the second highest contributor for point E (2.37%). Side D (2.62%) and side A (1.66%) contributed the least to points D and E, respectively. No contributions from side B and C were observed at receptor points D and E.

#### 3.2.2. Day 4 Afternoon

On day 4, the afternoon wind direction varied between 337.84° (NNW) and 315.75° (NNW) during sampling. Similarly to day 3, the predominant radon contributor was side A at all receptor points and the overall concentration decreased with increasing distance and divergence from the source’s downwind direction.

At receptor point A, only three (3) sides contributed towards radon concentration at this receptor. Side A was the largest contributor (77.25%), followed by side C (21.5%), and finally side D (1.25%). At receptor B, side A’s contribution decreased from 77.25% at receptor A to 66.40% while side C’s contribution was the second highest at 30.58%. Side D with the third highest at 2.96% and the smallest contributor being side B with 0.06%. Side E only contributed at receptor C and D (0.01% and 0.12%, respectively). At these two receptors, side A still dominated, followed by side D.

The downwind radon concentration is directly affected by orientation of the radon source and wind direction. It is assumed that the highest average predicted and observed concentrations are expected when the mean wind direction deviation angle from the source to the receptor is close to zero [46]. Furthermore, the source term is a function of the release area downwind to the receptors. The smaller the area source, the lower the total emission rates from the source and hence lower the radon concentration at the receptor point downwind. From these observations, it appears that the near source concentrations at the receptors directly downwind will, in the short term (1-h), be overestimated while receptors positioned at large deviation angles from the mean wind direction will be underestimated. Since side B has the smallest area compared to the other sides, its area source term would be the smallest, resulting in low day 4 concentrations. Accordingly, when all sides are directly in line with receptors downwind, it is envisaged that the radon contributions from this side will be minimal. Due to small area source term in line with mean wind direction, day 4 receptors had relatively low concentrations compared to day 3.

### 3.3. Accounting for Wake Effects

For each of the ISCST3 model runs described in Table 6, a complementary 1-h model run of ISC_PRIME was conducted to compute additional atmospheric dispersion due to the wake effects. The results of the wake modelling runs and the ISC-PRIME to ISC3ST concentration ratios are presented in Table 11 and Table 12, respectively.

The 1-h ISC-PRIME to ISCST3 average radon concentration ratios, which indicate the increase in radon concentration due to wake effects ranged from 1.00 at remote distances from the tailings dam to 2.39 at relatively close distances to the source, as shown in Table 13. The highest ratio of 2.39 was found on the lee side of the tailings dam, very close to the dam for the top-level area source. This is the near wake recirculation or cavity zone, which is characterized by significantly reduced wind speeds and intensive turbulence leading to rapid mixing [47]. The plume becomes trapped in the cavity, leading to increased concentrations very close to the leeward face of the dam. In this region, the effects of the wakes are very significant.

Beyond 250 m the distance, the far-wake turbulence zone, the highest ratios observed were 1.740 (top level area), 1.159 (true geometry area), 1.148 (ground level area) and 1.114 (volume source) at distances of 272.5 m, 272 m, 521 m and 521 m, respectively. The far-wake zone is very unsteady, and the airflow streamlines affect the wind speed and turbulence [47]. Further downwind of the tailings, in the PRIME calculated fields of turbulence intensity, the slopes of the streamlines and wind speeds as a function of the shape of the projected tailings dam will gradually decay to atmospheric values and the ratio approaches 1. This was particularly evident during the late mornings when the temperatures and the wind speed were gradually increasing.

Another finding was that, as compared to other modelling scenarios, the near-ground concentrations without wakes for top level area modelling were generally the lowest, as shown in Table 6 above. When the wakes effect was taken into consideration, the radon concentrations from the tailings dam increased by up to 239 percent for the top-level area source geometry. This is a very significant increase that was previously unaccounted for in the literature for radon modelling studies from tailings dams.

Due to the complexity of the ISCST-PRIME model, the inconsistent and convoluted results in Figure 11 should not be surprising. The radon concentrations predicted by the ratios in Table 12 are highly influenced by a combination of tailings geometry, climatic conditions affecting the plume rise, and thus wake specifications and cavity dimensions [48].

### 3.4. Measured vs. Modeled Radon Concentrations

**Figure 12:**
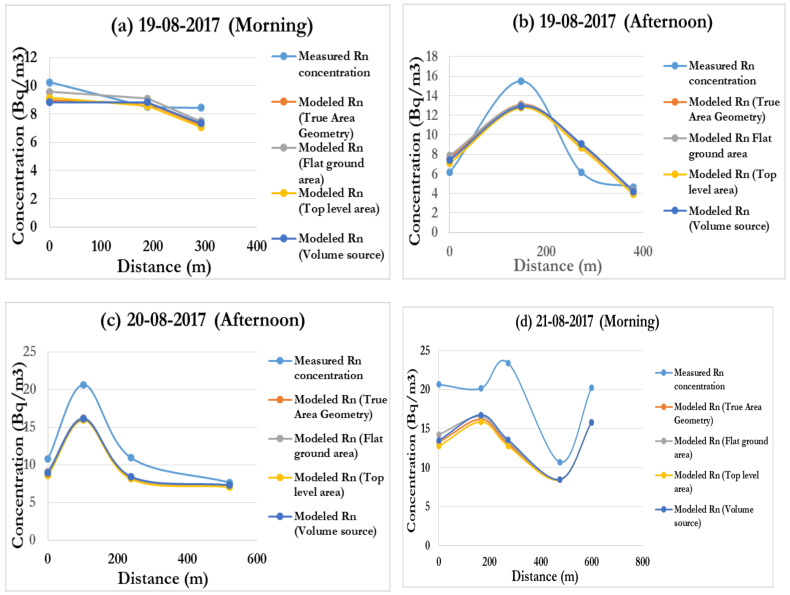

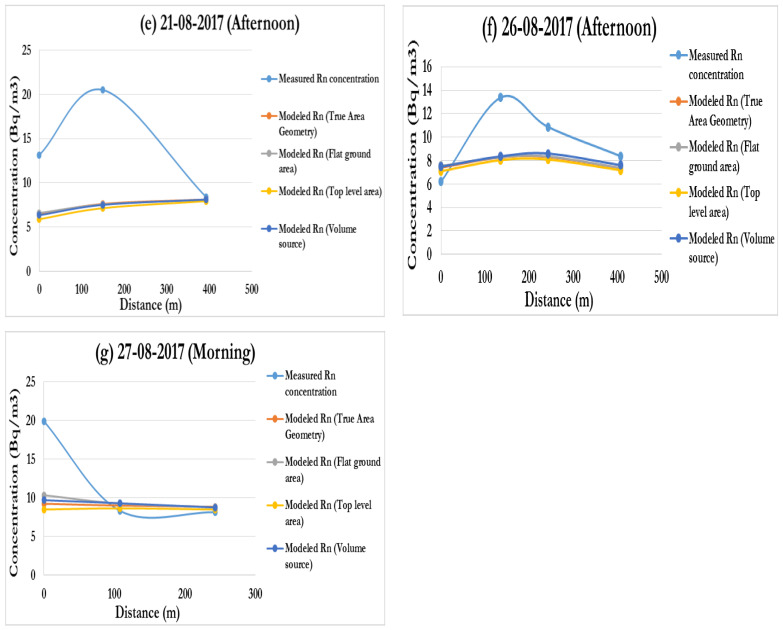
(**a**–**g**). Measured and modelled concentrations (background included).

The results of the measured radon concentrations for both downwind and upwind are presented in Table 13 and comparisons between these measured values and the wakes incorporated modelled concentrations are graphically presented in Figure 12a–g.

In terms of various peaks and dips, the graphical patterns in Figure 12a–g demonstrate that measured and modelled curves follow a similar trend. Except for (e), (f), and to some extent (g), there appears to be good agreement between measured and modelled radon in terms of radon distribution trend at various distances from the source in all these cases. The curves deviate significantly at two locations in cases (e) and (f), but only at one point in case (g). Surprisingly, when compensated for wake effects, all of the source geometries appear to provide nearly same results.

Common to all of the results presented in Figure 12 is that around the area close to the tailings, at distances in the region of 100–150 m downwind, the tailings position, dimension and exit parameters have a very substantial effect in radon distribution in that region [24]. As a result of the complicated spatial distribution of the concentration field around this region, both modelled and measured concentrations deviate from the linear dependency of the total release inventory. Within this distance, variations of both the wind speed and temperature follow the same sensitivity trend with distance from the tailings source. The peaks around this area for both morning and afternoon runs confirm this trend, which is consistent with the observations by [23].

At distances greater than 200 m from the tailings, the measured and modelled concentrations patterns agree with the theoretical findings which exhibit an inverse relationship between the pollutant concentration and the downwind distance from the source [49]. In this instance, the source dimension becomes less important, and the modelled and measured concentrations show linear dependence, thereby levelling out [50].

## 4. Conclusions

The ISCST3 code was used to evaluate radon transport and the effects of local variations of conditions around the tailings dam. This study was aimed at improving the accuracy and reliability of the dispersion modelling by focussing mainly on the source term. The source term was corrected to account for cracks and fissures caused by erosion on the side of the tailings dam walls. In addition, the geometry of the tailings dam was modelled by considering all emitting surfaces as source elements. This improved the accuracy of the model predictions as compared to traditional approaches to modelling source terms from tailings dams. As a result, the corrected emission rates were more realistic and presented accurate representations of the true source term. While the top-level area source term produced lower concentrations at near-field ground level receptors without wakes. incorporating the wakes effect increased the radon concentrations from tailings dam by up to 239% for the top-level area source. This result is a strong indication of the necessity to include wake calculations in modelling to accurately estimate radon exposure from the tailings dam. In addition, the methods used in this study can be applied to other approved gaussian models such as AERMOD to accurately predict radon dispersion from tailings dam and other similar structures. The performance of the model was validated by comparing the modelled results with the measured values. The results showed a good agreement between modelled concentration and measured concentrations, particularly for wake incorporated true geometry modelling scenario.

From modelling results, the radon concentrations from the tailings at the receptors were found to be significantly below the action level of 100 Bq/m^3^ as per National Nuclear Regulator Act 47 of 1999. The model predicted the highest concentration of 0.84 Bq/m^3^ at the receptor point from the true geometry source, which correspond to a dose of 0.012 mSv/y to the public due to radon from the tailings. This value is less than the 1 mSv/y dose constraint stipulated by the National Nuclear Regulator. It should however be noted that it was not the intention of this study to estimate highest dose values or the impact of the dam thereof. These values are mentioned as an aside.

Consequently, potential adverse environmental influences due to radon from the tailings can be simulated with an increased degree of accuracy. In South Africa, the close proximity of the mine tailings to the residential areas may pose some health risks. The results of this study will serve as a useful tool for assessing the radiological impact for regulatory purposes.

## Figures and Tables

**Table 1:** Day 1 input meteorological ASCII file.

0364300			2017	0364300			2017	
17	819	1	241.000	5.5200	279.5	4	800.0	800.0
17	819	2	246.000	5.6800	279.2	4	800.0	800.0
17	819	3	245.000	5.5150	278.9	4	800.0	800.0
17	819	4	250.000	4.9550	278.3	4	800.0	800.0
17	819	5	259.000	4.3900	277.7	4	800.0	800.0
17	819	6	262.000	4.2900	277.3	5	410.9	410.9
17	819	7	260.000	4.1900	277.0	5	406.0	406.0
17	819	8	228.000	2.6950	277.5	3	1000.0	1000.0
17	819	9	219.000	2.9500	278.7	4	800.0	800.0
17	819	10	223.000	4.9500	280.8	4	800.0	800.0
17	819	11	219.000	5.2800	282.7	4	800.0	800.0
17	819	12	207.000	5.3350	284.9	4	800.0	800.0
17	819	13	209.000	5.0450	286.9	3	1000.0	1000.0
17	819	14	211.000	3.0630	288.9	1	1800.0	1800.0
17	819	15	216.000	2.7000	289.9	2	1400.0	1400.0
17	819	16	228.000	2.3600	290.4	2	1400.0	1400.0
17	819	17	221.000	2.5650	290.2	3	1000.0	1000.0
17	819	18	221.000	3.0050	289.3	5	343.9	343.9
17	819	19	211.000	3.0400	287.0	6	191.8	191.8
17	819	20	221.000	3.1300	284.6	6	194,7	194.7
17	819	21	270.000	2.9900	282.9	6	190.3	190.3
17	819	22	264.000	3.9450	282.6	5	394.0	394.0
17	819	23	255.000	5.9450	282.3	4	800.0	800.0
17	819	24	55.000	6.5250	282.0	5	506.7	506.7

**Table 2:** Corrected side view surface measurements.

Tailing Side	Tape Length (m)	String Length (m)	Correction Factor (String/Tape)
Side A	2.00	2.45	1.23
Side B	2.00	2.48	1.24
Side C	2.00	2.54	1.27
Side D	2.00	2.63	1.32
Side E	2.00	2.47	1.24
Average	2.00	2.51	1.26

**Table 3:** Virtual point sources’ emission rates for each side of the tailings.

Side	Area Flux (Bq s^−1^ m^−2^)	Area (Google Earth) (m^2^)	Virtual Point Emission Rate (Bq s^−1^)
A	0.102	1.20×10^5^	1.22×10 ^4^
B	0.102	2.18 ×10 ^4^	2.23×10 ^3^
C	0.102	1.28×10 ^5^	1.30×10 ^4^
D	0.102	1.07×10 ^5^	1.09×10 ^4^
E	0.102	7.04×10 ^4^	7.15×10 ^3^

**Table 4:** Pasquil stability dependent coefficients.

Pasquill Stability Category	*p*	*q*
A (1)	209	0.89
B (2)	155	0.90
C (3)	103	0.92
D (4)	68	0.92
E (5)	51	0.92
F (6)	34	0.92

**Table 5:** Day 2 afternoon lateral distances and emission heights.

Hour	Pasquill Stability Category	Side Length, S (m)	$\mathrm{Standard}\;\mathrm{Deviation},\;\sigma_{y0}\;(m)$	$\sigma_{y0}/p\;(m)$	1/q	Lateral Virtual Distance *x_y_* (km)	Lateral Virtual Distance *x_y_* (m)	Emission Height (m)
**Side A**								
13	2	346	80.52	0.52	1.11	0.49	485.68	14
14	1	346	80.52	0.39	1.12	0.34	342.16	14
15	2	346	80.52	0.52	1.11	0.49	485.68	14
16	2	346	80.52	0.52	1.11	0.49	485.68	14
**Side B**								
13	2	148	34.36	0.22	1.11	0.19	188.94	75
14	1	148	34.36	0.16	1.12	0.13	131.42	75
15	2	148	34.36	0.22	1.11	0.19	188.94	75
16	2	148	34.36	0.22	1.11	0.19	188.94	75
**Side C**								
13	2	357	83.04	0.54	1.11	0.50	502.56	75
14	1	357	83.04	0.40	1.12	0.35	354.22	75
15	2	357	83.04	0.54	1.11	0.50	502.56	75
16	2	357	83.04	0.54	1.11	0.50	502.56	75
**Side D**								
13	2	328	76.22	0.49	1.11	0.46	457.01	75
14	1	328	76.22	0.36	1.12	0.32	321.70	75
15	2	328	76.22	0.49	1.11	0.46	457.01	75
16	2	328	76.22	0.49	1.11	0.46	457.01	75
**Side E**								
13	2	265	61.71	0.40	1.11	0.36	362	14
14	1	265	61.71	0.30	1.12	0.25	254	14
15	2	265	61.71	0.40	1.11	0.36	362	14
16	2	265	61.71	0.40	1.11	0.36	362	14

**Table 6:** Modelled 1-h ISCST3 concentrations—No wakes.

Date/Time	Receptor Point	Co-Ordinates	Modelled Rn Concentrations (Bq/m^3^)			
			True Geometry	Flat Ground-Level Area	Top Level Area	Volume Source
**19-08-2017: Day 1 morning**						
09:30	A	27°50′11″ S, 26°40′1″ E	0.51	1.10	0.07	0.39
11:12	B	27°50′16″ S, 26°39′57″ E	0.33	0.65	0.13	0.40
12:00	C	27°50′18″ S, 26°39′54″ E	0.33	0.57	0.15	0.43
**Day 1 afternoon**						
13:32	A	27°50′11″ S, 26°40′4″ E	0.73	0.94	0.18	0.50
14:30	B	27°50′15″ S, 26°40′1″ E	0.42	0.36	0.084	0.23
15:26	C	27°50′18″ S, 26°39′58″ E	0.49	0.56	0.21	0.66
16:12	D	27°50′21″ S, 26°39′56”E	0.47	0.54	0.12	0.40
**20-08-2017: Day 2 afternoon**						
13:08	A	27°50′11″ S, 26°40′10″ E	0.53	0.68	0.12	0.51
13:54	B	27°50′13″ S, 26°40′7″ E	0.38	0.40	0.15	0.31
14:50	C	27°50′17″ S, 26°40′9″ E	0.44	0.47	0.22	0.54
15:49	D	27°50′22″ S, 26°40′5″ E	0.32	0.31	0.16	0.40
**21-08-2017: Day 3 morning**						
07:39	A	27°50′11″ S, 26°40′0″ E	0.56	1.50	5.3×10 ^−2^	0.79
08:35	B	27°50′15″ S, 26°39′56″ E	0.39	0.76	5.1×10 ^−2^	0.94
09:25	C	27°50′18″ S, 26°39′54″ E	0.35	0.60	6.5×10 ^−2^	0.84
10:28	D	27°50′24″ S, 26°39′51″ E	1.1×10 ^−2^	9.5×10 ^−3^	5.4×10 ^−6^	3.4×10 ^−2^
11:14	E	27°50′28″ S, 26°39′51″ E	8.5×10 ^−5^	6.8×10 ^−5^	0	7.6×10 ^−3^
**Day 3 afternoon**						
13:00	A	27°50′10″ S, 26°40′22″ E	0.75	0.91	0.21	0.65
13:52	B	27°50′12″ S, 26°40′27″ E	0.62	0.57	0.14	0.51
15:24	C	27°50′15″ S, 26°40′35″ E	0.22	0.18	3.6×10 ^−2^	0.22
**26-08-2017: Day 4 afternoon**						
13:19	A	27°50′11″ S, 26°40′18″ E	0.41	0.55	0.10	0.48
14:12	B	27°50′15″ S, 26°40′20″ E	0.37	0.39	0.14	0.46
15:04	C	27°50′18″ S, 26°40′22″ E	0.41	0.46	0.20	0.71
16:04	D	27°50′22″ S, 26°40′26″ E	0.37	0.39	0.19	0.62
**27-08-2017: Day 5 morning**						
08:02	A	27°50′11″ S, 26°40′4″ E	0.79	1.90	4.9×10 ^−2^	1.2
08:50	B	27°50′14″ S, 26°40′2″ E	0.55	0.81	0.20	0.85
09:43	C	27°50′18″ S, 26°40′0″ E	0.40	0.34	4.3	0.30

**Table 7:** Individual side concentrations (day 3).

Receptor Point	Co-Ordinates	Side A (Bq m^−3^)	Side B (Bq m^−3^)	Side C (Bq m^−3^)	Side D (Bq m^−3^)	Side E (Bq m^−3^)	Total (Bq m^−3^)
A	27°50′11″ S, 26°40′0″ E	0.41	0	8.6×10 ^−2^	6.0×10 ^−4^	0	0.46
B	27°50′15″ S, 26°39′56″ E	0.19	0	9.0×10 ^−5^	0.11	0	0.30
C	27°50′18″ S, 26°39′54″ E	0.15	0	3.5×10 ^−4^	0.10	0	0.25
D	27°50′24″ S, 26°39′51″ E	5.8×10 ^−4^	0	0	2.9×10 ^−4^	0.01	0.01
E	27°50′28″ S, 26°39′51″ E	1.4×10 ^−6^	0	0	2.0×10 ^−6^	8.1×10 ^−5^	8.4×10 ^−5^

**Table 8:** Percentage contribution by each side (day 3).

Receptor Point	Co-Ordinates	Side A (%)	Side B (%)	Side C (%)	Side D (%)	Side E (%)	Total (%)
A	27°50′11″ S, 26°40′0″ E	81	0	18.97	0.13	0	100
B	27°50′15″ S, 26°39′56″ E	64.7	0	0.03	35.27	0	100
C	27°50′18″ S, 26°39′54″ E	60.46	0	0.14	39.4	0	100
D	27°50′24″ S, 26°39′51″ E	5.24	0	0	2.62	92.14	100
E	27°50′28″ S, 26°39′51″ E	1.66	0	0	2.37	95.97	100

**Table 9:** Individual side concentrations (day 4).

Receptor Point	Co-Ordinates	Side A (Bq m^−3^)	Side B (Bq m^−3^)	Side C (Bsq m^−3^)	Side D (Bq m^−3^)	Side E (Bq m^−3^)	Total (Bq m^−3^)
A	27°50′11″ S, 26°40′18″ E	0.22	0	0.06	3.6×10 ^−3^	0	0.29
B	27°50′15″ S, 26°40′20″ E	0.14	1.2×10 ^−4^	0.07	6.3×10 ^−3^	0	0.21
C	27°50′18″ S, 26°40′22″ E	0.14	0	8×10 ^−3^	0.041	2.1×10 ^−5^	0.19
D	27°50′22″ S, 26°40′26″ E	0.11	9.5×10 ^−5^	0.01	0.039	1.8×10 ^−4^	0.16

**Table 10:** Percentage contribution by each side (day 4).

Receptor Point	Co-Ordinates	Side A (%)	Side B (%)	Side C (%)	Side D (%)	Side E (%)	Total (%)
A	27°50′11″ S, 26°40′18″ E	77.25	0	21.5	1.25	0	100
B	27°50′15″ S, 26°40′20″ E	66.40	0.06	30.58	2.96	0	100
C	27°50′18″ S, 26°40′22″ E	73.67	0	4.42	21.90	0.01	100
D	27°50′22″ S, 26°40′26″ E	69.33	0.06	6.29	24.20	0.12	100

**Table 11:** Wake modelling runs.

Date/Time	Receptor Point	Co-Ordinates	Modelled Rn Concentrations (Bq m^−3^)			
			True Geometry	Flat Ground Area	Top Level Area	Volume Source
**19-08-2017 Morning**						
09:30	A	27°50′11″ S, 26°40′1″ E	0.574	1.189	0.766	0.458
11:12	B	27°50′16″ S, 26°39′57″ E	0.326	0.648	0.128	0.396
12:00	C	27°50′18″ S, 26°39′54″ E	0.337	0.573	0.156	0.437
**Afternoon**						
13:32	A	27°50′11″ S, 26°40′4″ E	0.821	1.032	0.262	0.583
14:30	B	27°50′15″ S, 26°40′1″ E	0.436	0.373	0.097	0.245
15:26	C	27°50′18″ S, 26°39′58″ E	0.570	0.640	0.293	0.735
16:12	D	27°50′21″ S, 26°39′56″ E	0.472	0.541	0.119	0.405
**20-08-2017 Afternoon**						
13:08	A	27°50′11″ S, 26°40′10″ E	0.575	0.719	0.164	0.548
13:54	B	27°50′13″ S, 26°40′7″ E	0.392	0.414	0.161	0.316
14:50	C	27°50′17″ S, 26°40′9″ E	0.472	0.498	0.252	0.573
15:49	D	27°50′22″ S, 26°40′5″ E	0.367	0.355	0.208	0.446
**21-08-2017 Morning**						
07:39	A	27°50′′11″ S, 26°40′0″ E	0.566	1.542	0.057	0.792
08:35	B	27°50′15″ S, 26°39′56″ E	0.433	0.809	0.097	0.989
09:25	C	27°50′18″ S, 26°39′54″ E	0.402	0.644	0.113	0.889
10:28	D	27°50′24″ S, 26°39′51″ E	0.012	0.010	5.43E-6	0.034
11:14	E	27°50′28″ S, 26°39′51″ E	0.000	0.000	0.000	0.008
**Afternoon**						
13:00	A	27°50′10″ S, 26°40′22″ E	0.796	0.956	0.252	0.698
13:52	B	27°50′12″ S, 26°40′27″ E	0.678	0.623	0.192	0.561
15:24	C	27°50′15″ S, 26°40′35″ E	0.232	0.194	0.046	0.228
**26-08-2017 Afternoon**						
13:19	A	27°50′11″ S, 26°40′18″ E	0.559	0.692	0.249	0.623
14:12	B	27°50′15″ S, 26°40′20″ E	0.449	0.462	0.212	0.536
15:04	C	27°50′18″ S, 26°40′22″ E	0.422	0.477	0.213	0.722
16:04	D	27°50′22″ S, 26°40′26″ E	0.382	0.397	0.203	0.630
**27-08-2017 Morning**						
08:02	A	27°50′11″ S, 26°40′4″ E	0.843	1.949	0.103	1.307
08:50	B	27°50′14″ S, 26°40′2″ E	0.589	0.849	0.229	0.882
09:43	C	27°50′18″ S, 26°40′0″ E	0.398	0.340	0.043	0.303

**Table 12:** ISC-PRIME to ISC3ST concentration ratios.

Date	Receptor Points	Distance (m)	ISC-PRIME to ISC3ST Concentration Ratios			
			True Geometry Area	Flat Ground Area	Top Level Area	Volume Source
**19-08-2017 Morning**						
09:30	A	0	1.129	1.058	1.093	1.167
11:12	B	189.2	1.001	1.001	1.003	1.001
12:00	C	291.8	1.008	1.004	1.016	1.006
**Afternoon**						
13:32	A	0	1.118	1.092	1.497	1.175
14:30	B	148.3	1.029	1.034	1.146	1.053
15:26	C	272.0	1.159	1.139	1.364	1.119
16:12	D	379.6	1.007	1.006	1.029	1.008
**20-08-2017 Afternoon**						
13:08	A	0	1.075	1.059	1.325	1.079
13:54	B	102.6	1.027	1.026	1.068	1.034
14:50	C	237.7	1.074	1.070	1.148	1.060
15:49	D	521.0	1.142	1.148	1.281	1.114
**21-08-2017 Morning**						
07:39	A	0	1.006	1.002	1.067	1.005
08:35	B	164.9	1.120	1.061	1.909	1.049
09:25	C	272.5	1.135	1.080	1.740	1.057
10:28	D	475.1	1.019	1.022	1.000	1.006
11:14	E	598.6	1.000	1.000	1.000	1.000
**Afternoon**						
13:00	A	0	1.062	1.051	1.228	1.072
13:52	B	149.9	1.085	1.093	1.383	1.105
15:24	C	392.5	1.044	1.053	1.267	1.045
**26-08-2017 Afternoon**						
13:19	A	0	1.349	1.264	2.391	1.303
14:12	B	135.1	1.203	1.196	1.558	1.164
15:04	C	242.7	1.033	1.029	1.068	1.019
16:04	D	407.6	1.028	1.027	1.054	1.017
**27-08-2017 Morning**						
08:02	A	0	1.069	1.029	2.123	1.044
08:50	B	107.6	1.062	1.042	1.175	1.040
09:43	C	242.7	1.000	1.000	1.000	1.000

**Table 13:** Measured downwind and background radon concentrations.

Day/Time	Receptor Points	Distance (m)	Measured Rn Concentration (Downwind) (Bq/m^3^)	Background Rn Concentration (Upwind) (Bq/m^3^)
**19-08-2017 Morning**				
09:30	A	0	10.3	8.437
11:12	B	189.2	8.5	8.437
12:00	C	291.8	8.4	6.907
**Afternoon**				
13:32	A	0	6.1	6.906
14:30	B	148.3	15.5	12.675
15:26	C	272.0	6.2	8.432
16:12	D	379.6	4.6	3.796
**20-08-2017 Afternoon**				
13:08	A	0	10.8	8.436
13:54	B	102.6	20.6	15.787
14:50	C	237.7	10.9	7.902
15:49	D	521	7.7	6.905
**21-08-2017 Morning**				
07:39	A	0	20.6	12.703
08:35	B	164.9	20.1	15.799
09:25	C	272.5	23.4	12.682
10:28	D	475.1	10.7	8.423
11:14	E	598.6	20.3	15.776
**Afternoon**				
13:00	A	0	13.1	5.678
13:52	B	149.9	20.5	6.991
15:24	C	392.5	8.4	7.891
**26-08-2017 Afternoon**				
13:19	A	0	6.2	6.996
14:12	B	135.1	13.4	7.903
15:04	C	242.7	10.9	7.895
16:04	D	407.6	8.38	6.993
**27-08-2017 Morning**				
08:02	A	0	19.9	8.430
08:50	B	107.6	8.3	8.432
09:43	C	242.7	8.1	8.433

## Data Availability

Additional data are available on request from the corresponding author.
